# Relationships between Spirituality, Happiness, and Prosocial Bystander Behavior in Bullying—The Mediating Role of Altruism

**DOI:** 10.3390/ejihpe12120128

**Published:** 2022-12-06

**Authors:** Fernanda Inéz García-Vázquez, Maria Fernanda Durón-Ramos, Rubén Pérez-Rios, Ricardo Ernesto Pérez-Ibarra

**Affiliations:** 1 Instituto Tecnológico de Sonora, Department of Education, Obregon 85000, Mexico; 2 Instituto Tecnológico de Sonora, Department of Guaymas, Guaymas 85400, Mexico

**Keywords:** bullying, prosocial bystander, spirituality, happiness, altruism

## Abstract

Positive psychology is vital in increasing prosocial behavior and reducing bullying. However, limited studies have analyzed the influence of positive personal characteristics on the prosocial behaviors of bystanders in bullying. The present study examined direct and indirect relationships between spirituality, happiness, altruism, and prosocial bystander behavior in bullying. Participants in this study were 685 students from Northwestern Mexico; 51% were male and 49% female, between 12 and 18 years old (*M* = 14.3 years, *SD* = 1.68). A structural equation model (SEM) was calculated. The results indicate that happiness and altruism were related to prosocial bystander behavior. Spirituality and happiness have an indirect relationship by increasing prosocial bystander behavior through the positive effects of altruism. The SEM explained 48% of the variance of the prosocial bystander. The implications for improving defensive behavior in bullying and reducing school violence are discussed.

## 1. Introduction

Bullying is a prevalent problem at all educational levels, with severe consequences for the participants [1,2,3]. This problem affects the psychosocial development of the students involved [4,5,6,7]. Bullying comprises repeated aggressive behavior toward peers who experience difficulty defending themselves [8,9]. The students involved participate as aggressors, victims, or bystanders [10,11]. Bystanders are students witnessing aggression without directly participating as aggressors or victims, and have a crucial role in bullying [12,13,14]. Some authors identify prosocial bystander behaviors in bullying [15,16]. These actors intervene in two ways: directly defending by stopping the aggression or indirectly by informing an adult or comforting the victims [15,17].

Some studies indicate that the prosocial bystander contributes to the reduction of bullying [18,19], increasing positive feelings and reducing depression, social anxiety, and loneliness, compared to those who have not been defended in victimization [20].

The research focused on prosocial bystanders highlighting the importance of individual characteristics to understand and explain their behavior [21,22,23,24]. The theory of bystanders in bullying emphasizes the role of personal determinants for understanding the underpinning factors that promote the development of prosocial behaviors in bystanders; for example, Ettekal et al. [21] highlight the attributes of students, specifically those related to their development, such as social-cognitive factors and emotions. Likewise, Meter and Card [25] emphasize the importance of analyzing individual factors and their influence on students deciding to defend their peers, together with interpersonal factors. 

In this regard, a considerable number of studies have focused on analyzing moral variables, such as moral emotions, moral judgment, and moral disengagement [26,27,28,29]. However, recent evidence suggests that a positive psychology approach is valuable in researching prosocial behaviors [30,31,32,33] and particularly in the study of prosocial bystander behavior in bullying [34,35]. Positive psychology emphasizes the study of character strengths such as spirituality; and positive experiences and behaviors, including happiness and altruism [36,37,38]. Therefore, the present study is aimed to explore the direct and indirect relationships between spirituality, happiness, altruism, and prosocial bystander behavior in Mexican adolescent students.

### 1.1. Spirituality

Spirituality is a human strength that involves beliefs and practices based on the conviction of the existence of a transcendent or non-physical dimension of life [39]. Some authors have stressed the relevance of spirituality to the development of prosocial behaviors [40,41]; for example, the positive effects of this strength on prosocial behaviors in adolescents have been reported [42,43]. Additionally, research shows that daily spiritual experiences are important predictors of prosocial behaviors [44]. 

In the bullying context, research indicates that university students reporting greater spiritual well-being were at a lower risk of victimization or bullying perpetration online [45]; additionally, in adolescence, spirituality is negatively associated with peer victimization and bullying [46,47]. 

### 1.2. Happiness

Happiness is a person’s subjective assessment of positive emotional experiences throughout life [48]. Some studies highlight how bullying negatively impacts the happiness of students [49,50]; however, it is important to address how happiness is related to bullying prevention by reducing aggressive behaviors [51,52] and increasing prosocial bystanders’ behavior in bullying [34]. Ample evidence suggests that happiness is positively related to prosocial behaviors [53,54,55,56]. A study on adolescents examined the effect of happiness on prosocial behavior online and found that positive emotional states act on happiness and promote prosocial behavior [57].

A substantial body of research indicates that happiness is related to reducing different types of aggression; for example, evidence indicates that happiness is negatively associated with reactive aggression [58]. Additionally, some studies with children and adolescents found that general happiness and happiness in school were negatively associated with aggression in bullying and cyberbullying [49,59].

### 1.3. The Mediating Role of Altruism

Altruism is considered a specific type of prosocial behavior [60,61,62,63], which refers to voluntary attitudes and commitment to help and attend to the needs of others without expecting rewards or direct benefits; in addition to being able to embrace the cost or sacrifice [61,64,65].

Some authors indicate altruism is a variable strongly related to spirituality [66,67,68,69]. For example, in a study, spiritual experiences and spiritual cognitions were strongly linked to altruism; furthermore, spirituality potently predicts altruism [66]. In another study, evidence showed that spirituality predicts greater compassion and altruism [69].

On the other hand, altruism is related to positive subjective experiences; a meta-analysis performed by Curry et al. [70] indicated that well-being might be improved by performing acts of kindness. In addition, the literature evidenced a positive relationship between positive emotions and positive effects on altruism [32,71]; for example, in a study exploring the relationships between happiness and altruism, evidence showed a significant association [72]. 

In the bullying context, altruistic motivation in bullying situations is related to prosocial behavior [73]. Additionally, altruistic behavior is associated with defense in the bystanders of homophobic behavior, finding more active participation in students with altruistic qualities [74]. Finally, a positive relationship was found between altruism and the willingness to intervene in bullying situations [75].

### 1.4. The Present Study

A substantial body of studies that address prosocial bystander behavior is focused on a moral theoretical perspective, such as moral emotions [27,76,77]. However, evidence suggests that the positive psychology approach, which emphasizes the study of human strengths and positive variables, is valuable in the study of prosocial behaviors [42,57,73]. Nonetheless, no study known by the authors explores the relationship between spirituality, altruism, happiness, and prosocial bystander behavior in bullying. In addition, the research in the field is mainly focused on antisocial behavior instead of prosocial behavior [78,79]; and a limited number of studies have examined the prosocial behavior in the bullying context [35]. Finally, the study of bystanders in Mexican students is incipient. Therefore, the present study is aimed to explore the direct and indirect relationships between spirituality, happiness, altruism, and prosocial bystander behavior in Mexican adolescent students.

## 2. Methods

### 2.1. Participants and Procedure

#### 2.1.1. Participants

This study included 685 students from eight public schools in one northern state of Mexico. Four schools had students from grades 7 to 9, while the other four contained pupils from grades 10 to 12, corresponding to Mexico’s second and third basic levels of education. In all, 51% of the students were male, while 49% were female. Ages ranged from 12 to 18 years old, with a mean of 14.3 (*SD* = 1.68). 

#### 2.1.2. Procedure

The university’s ethics committee, where the authors are affiliated, approved the research project (official letter 142). All the school’s principals agreed to perform the study. Then, the parents were informed through a meeting, and requested their approval for the minor’s participation. Only 2% denied the authorization. Finally, a written self-report was presented to the students, explaining the anonymous and volunteer participation, and all participants agreed to be included in the research.

A database compatible with SPSS and AMOS was created with participants’ responses. First, the authors performed a Confirmatory Factor Analysis (CFA) to establish the validity of each scale. The goodness of fit indices considered was X^2^, Standardized Root Mean Squared Residual (SRMR), adjusted goodness of fit index (AGFI), Tucker-Lewis Index (TLI), and Root Mean Square Error of Approximation (RMSEA) [80]. Second, the internal consistency of each scale was obtained through Cronbach’s Alpha. Finally, a Structural Equation Model (SEM) was constructed to analyze direct relationships between the variables included in the study using the AMOS software. The bootstrap method was used with 500 repetitions and a 95% confidence interval. 

### 2.2. Measures

The instrument included: (a) informed consent, (b) demographic characteristics (gender, age, and grade), and (c) four scales to measure the variables of interest:

#### 2.2.1. Spirituality

A scale was developed combining items from the Character Strengths Inventory for Children, CSI-C [81], and Values in Action Inventory of Strengths for Youth, VIA-Youth [82]. This adaptation was performed to fit the population of this study (early and middle adolescents); scale was composed of four items (e.g., I love and like spiritual things, like praying, doing techniques to develop the imagination, or breathing and relaxation techniques). Participants could respond using a Likert scale from 1 (totally disagree) to 5 (totally agree). Internal consistency for the scale was acceptable with a Cronbach alpha of 0.84. A Confirmatory Factor Analysis (CFA) was performed to obtain the validity of the scale; CFA presented acceptable indicators of goodness of fit (X^2^ = 1.203, *df* = 2, *p* = 0.548; SRMR = 0.01; AGFI = 0.99; TLI = 1.00; CFI = 1.00; RMSEA = 0.00, CI 90 [0.00, 0.06]).

#### 2.2.2. Happiness

An adaptation of the Oxford Happiness Inventory [83] was used. The scale utilizes six items (e.g., I am very happy). Participants could respond using a Likert scale from 1 (totally disagree) to 5 (totally agree). Cronbach Alpha was 0.77, indicating the internal consistency for the scale. Confirmatory Factor Analysis presented acceptable indicators of goodness of fit. (X^2^ = 5.906, *df* = 9, *p* = 0.749; SRMR = 0.01; AGFI = 0.99; TLI = 1.00; CFI = 1.00; RMSEA = 0.00, CI 90 (0.00, 0.03)), indicating the validity of the scale.

#### 2.2.3. Altruism

The Generative Altruism Scale [65] was used. This scale was composed of seven items (e.g., I help others even when there is no direct benefit to me). Participants could respond using a Likert scale from 1 (never) to 5 (always). Internal consistency for the scale was acceptable with a Cronbach alpha of 0.70. A Confirmatory Factor Analysis (CFA) was performed to obtain the validity of the scale; CFA presented acceptable indicators of goodness of fit (X^2^ = 24.746, *df* = 13, *p* = 0.025; SRMR = 0.03, AGFI = 0.97; TLI = 0.97; CFI = 0.98; RMSEA = 0.03, IC 90 (0.01, 0.05)).

#### 2.2.4. Prosocial Bystander

An adaptation of the subscale from the Bullying Participant Behaviors Questionnaire [17] was used. The scale included seven items (e.g., I have defended a colleague who has been pushed, hit, or slapped). Participants could respond using a Likert scale from 1 (never) to 5 (always). Cronbach Alpha was 0.86, indicating the internal consistency for the scale. Confirmatory Factor Analysis presented acceptable indicators of goodness of fit (X^2^ = 15.056, *df* = 11, *p* = 0.180; SRMR = 0.03; AGFI = 0.98; TLI = 0.99; CFI = 0.99; RMSEA = 0.02, IC 90 (0.00, 0.04)), indicating the validity of the scale.

## 3. Results

Table 1 presents the measures of central tendency, the normality of data distribution (Skewness and Kurtosis), and the correlation matrix. According to the mean and standard deviation of the indices representing each variable, spirituality obtained a lower average (*M* = 2.90, *SD* = 1.12) compared to happiness, altruism, and the prosocial bystander. The data distribution was normal according to the skewness and kurtosis results, located between −1 and 1 [80]. Finally, the correlation matrix presented positive and significant associations (*p* < 0.001) between all the variables studied. 

**Figure 1:**
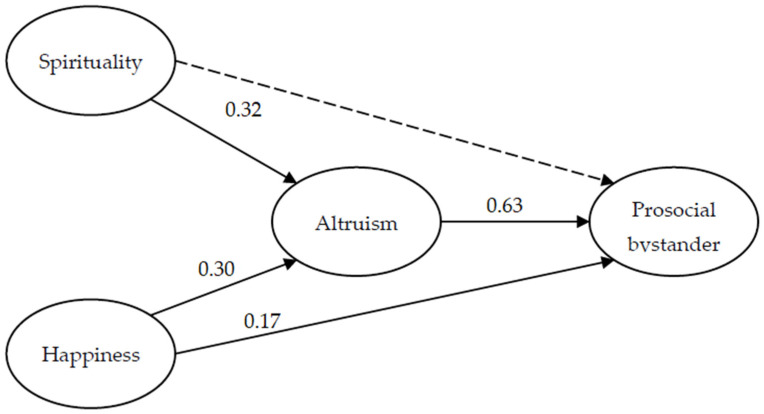
SEM direct and indirect effects on prosocial bystander from spirituality, happiness, and altruism.

The results of the structural equation model (SEM) are presented in Figure 1. The calculation of the SEM showed an acceptable fit to the data (χ^2^ = 387.22, *df* = 242, *p* = 0.000; SRMR = 0.05; AGFI = 0.94; CFI = 0.97; TLI = 0.97; RMSEA = 0.03, CI (0.02, 0.03)) and explained 48% of the variance in prosocial bystander. The direct effects results indicated that happiness is positively associated with altruism (β = 0.30, *p* < 0.000) and the prosocial bystander (β = 0.17, *p* < 0.000). Spirituality is positively associated with altruism (β = 0.32, *p* < 0.000), however, it was not related to the prosocial bystander (β = −0.06, *p* < 0.205). On the other hand, altruism was positively associated with the prosocial bystander (β = 0.63, *p* < 0.000).

Regarding the indirect effects, the results indicated that both happiness and spirituality (β = 0.19, CI (0.11, 0.26), *p* < 0.013; β = 0.20, CI (0.12, 0.28), *p* < 0.019, respectively) favor the prosocial bystander through its positive association with altruism.

## 4. Discussion

Bullying is a phenomenon that affects students’ health worldwide [84]; investigations focused on bullying in Mexico are not mainly focused on variables that could prevent bullying [85]. Therefore, this research proposes three positive factors, studied mainly in positive psychology, that could reduce the probability of bullying by enhancing the prosocial bystander’s behaviors, emphasizing altruism as a mediator between spirituality and happiness.

The structural equation model showed a positive relationship between spirituality and altruism, reinforcing that people with a higher sense of spirituality are more likely to perform altruistic behavior [66,69]. Spirituality does not present a significant direct relationship with prosocial bystanders; however, it is indirectly related through altruism. In a similar study presented by Li and Chow [42], the relationship between spirituality was related to peer-helping behaviors, and this relation was mediated by gratitude; however, spirituality and stranger-helping behavior did not present a significant association. This could indicate that spirituality and prosocial bystander behavior are related when (a) a third factor is present, such as altruism or gratitude, and (b) the victim is someone they do not consider a stranger. In fact, peer relationships are important when bullying appears [25].

Happiness presents a direct and significative relationship with altruism, indicating that happy people tend to perform giving or altruistic behavior [86]. The model also showed a direct and significant relationship between happiness and prosocial bystander behavior, which was also found previously [34]. The evidence provided suggests that happy students tend to act more altruistically, and if they witness bullying, they are more likely to help the victims.

Finally, according to our results, there is a direct and significant link between altruism and prosocial bystanders’ behavior; this relationship has been found in previous research focused on prosocial behavior [63,73,87]. Altruism is a variable that predicts the prosocial behavior of bystanders, increasing the probability of defending and helping victims of bullying [75].

When interpreting these results, some limitations should be considered. First, self-report scales were used in the study, which may lead to the social desirability of responses. Second, the sample included urban school students from a specific geographic location, making it difficult to generalize the findings. Therefore, we recommend carrying out studies with more extensive and diverse samples. Finally, the study has a cross-sectional design that does not allow for verifying the causal relationships between the variables in a strict sense. In this regard, we suggest studies with a longitudinal or experimental design. Regardless of these limitations, this study provides evidence of the direct and indirect relationship between positive factors and prosocial behavior from the bystanders of bullying.

Further investigations on bullying should focus on positive factors in adolescents, such as character strengths, to better understand how these variables can promote prosocial bystander behavior in bullying situations. It would be relevant to explore the role of virtues; for example, temperance [88] has proven to be valuable in studying different types of aggression, so it could also help to understand the adoption of prosocial roles in bystanders. In addition, exploring positive psychology variables, such as gratitude, forgiveness, courage, or optimism, could be fruitful in studying these behaviors. 

In addition, it would be relevant to study the situations in which altruism is more effective and leads to better results related to prosocial behavior, for example, if they consider the person as someone less fortunate [89]. A final consideration for future research in this matter is that prosocial bystander behavior should be studied considering the closeness with the victim.

## 5. Conclusions

The results provide evidence that bystanders of bullying could increase their prosocial behaviors by strengthening spirituality, altruism, and happiness. Therefore, it is essential to consider interventions aimed at promoting these positive factors in adolescents to contribute to the prevention of bullying.

## Figures and Tables

**Table 1:** Descriptive statistics and correlation matrix.

	M	SD	Skewness	Kurtosis	1	2	3	4
(1) Spirituality	2.90	1.12	0.039	−0.921	_			
(2) Happiness	3.52	0.81	−0.461	−0.071	0.25 **	_		
(3) Altruism	3.48	0.65	−0.181	−0.196	0.33 **	0.30 **	_	
(4) Prosocial bystander	3.41	0.94	−0.212	−0.681	0.22 **	0.32 **	0.49 **	_

** *p* < 0.001.

## Data Availability

Data supporting reported results can be found at: http://dx.doi.org/10.17632/nk2ghcdj4s.1 (accessed on 1 October 2022).
